# From sliding clamp to stationary repressor: how clamp-locking proteins convert KorB into an effective long-range gene silencer

**DOI:** 10.1042/BST20260153

**Published:** 2026-11-11

**Authors:** Thomas C. McLean

**Affiliations:** 1 University of Melbourne, Melbourne, VIC, Australia; 2 The Walter and Eliza Hall Institute of Medical Research, Parkville, VIC, Australia

**Keywords:** antibiotic resistance, gene expression and regulation, Plasmid biology

## Abstract

Transcriptional repression in prokaryotes is typically achieved by repressor proteins that bind close to, or within, the core promoters they control. KorB, a plasmid-partitioning protein essential for stable plasmid inheritance that additionally acts as a transcriptional repressor, poses a puzzle for this canonical view: it can control promoters located kilobase distances from its DNA binding site. This spatial discrepancy motivated three decades of competing models for KorB-mediated repression, and none of the emerging theories of polymerisation along DNA and/or long-range DNA looping proved entirely satisfactory. The present mini-review discusses recent work demonstrating that KorB instead solves this problem dynamically: it is a self-loading, CTP-dependent DNA-sliding clamp protein that entraps DNA and diffuses away from its loading site to reach distal target promoters. Acting alone, KorB is a poor repressor, confounded by the spatiotemporal limitations of 1D-diffusion. Effective, site-specific silencing instead relies on a second class of protein, exemplified by KorA and, more recently, the paralogous protein TrbA. These clamp-locking proteins lock the sliding KorB in place at target promoters, dramatically increasing repression. The present mini-review details the structural basis of this clamp-locking interaction, its consequences for transcriptional initiation, and the broader questions this sliding-clamp-to-repressor strategy raises for long-range gene regulation, plasmid biology, and the spread of antimicrobial resistance.

## Introduction

The commitment from DNA to RNA through the process of transcription is one of the most pivotal steps made by cells. This is particularly pronounced in prokaryotes, where transcription and translation are coupled and many post-transcriptional regulatory mechanisms available to eukaryotes are absent [1]. Consequently, transcriptional regulation, specifically repression, is an ancient and highly complex process capable of targeting multiple stages of transcriptional initiation and elongation [2–4].

Canonical prokaryotic repression mechanisms target RNA polymerase (RNAP) at distinct stages. Some repressors bind proximal to the core promoter and block polymerase access through steric hindrance, while others prevent the closed-to-open promoter complex transition. Additional mechanisms prevent polymerase escape during initiation, and downstream-binding repressors can similarly obstruct elongation (Figure 1A). These mechanisms have been extensively reviewed elsewhere [2,3]. Importantly, these processes share a unifying principle: all require the repressor to bind proximal to the core promoter or within the target gene. However, in numerous cases, transcriptional repression has been experimentally demonstrated even though the repressor's binding site maps far from the promoter, outside any canonical regulatory regions, implying a mechanism that diverges entirely from the classical model. How, then, can repression occur when a binding site is positioned too distantly to exert direct steric effects on promoter-proximal events?

**Figure 1:**
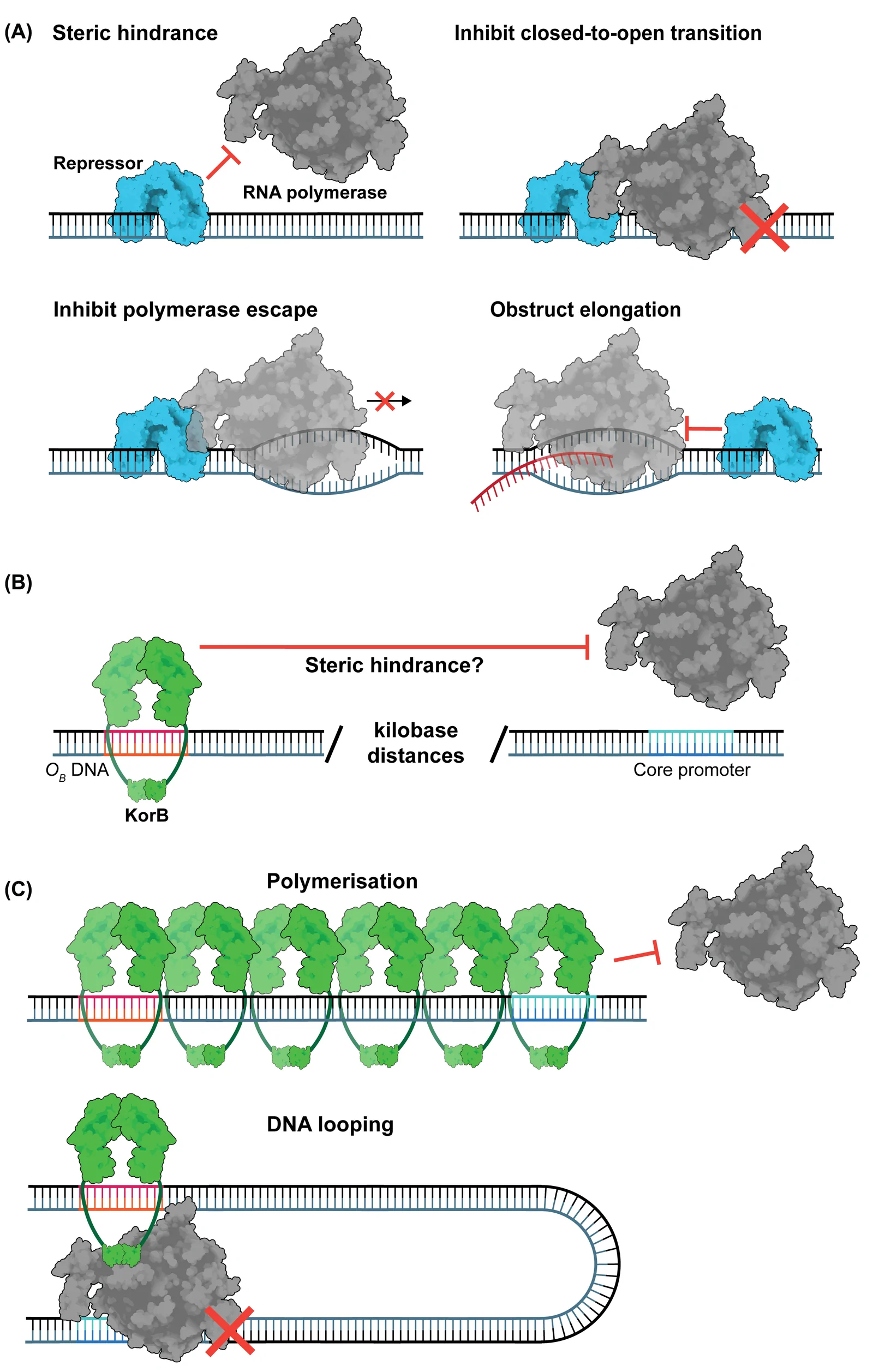
Canonical prokaryotic repression mechanisms cannot easily account for KorB-mediated silencing (**A**) Repressor proteins (cyan) can block transcription by RNAP (grey) through several canonical mechanisms: steric hindrance of RNAP binding to the core promoter, inhibition of the closed-to-open promoter complex transition, inhibition of RNAP escape from the promoter, or obstruction of RNAP during elongation. In each case, the repressor must bind directly at, or immediately adjacent to, the site of RNAP action. (**B**) KorB (green) instead binds its cognate operator, *O_B_*, which can be positioned kilobase distances from the core promoter it represses, far beyond the reach of a statically bound repressor. (**C**) Two models were proposed to reconcile this. In the first, KorB polymerises along the DNA from *O_B_* until it reaches and occludes the promoter; this is difficult to reconcile with estimates of cellular KorB abundance, which are an order of magnitude lower than those of ParB homologues that silence by this route. In the second, DNA looping brings a distally bound KorB into contact with RNAP at the promoter, preventing isomerisation from the closed to open complex rather than excluding RNAP entirely; however, repression is largely unaffected by moving *O_B_* to the opposite face of the DNA helix, which argues against a simple looping mechanism.

Recent advances in sequencing technologies, chromosome conformation capture, and single-molecule techniques have enabled the characterisation of long-range gene silencing mechanisms that prokaryotes employ to suppress transcription across extended chromosomal regions [5,6]. Throughout the present review, long-range repression refers to repression outside the canonical proximal window discussed above, where the repressor is too distant from the core promoter or target gene to interfere directly with RNAP. Repression from such a site must instead rely on a mechanism that propagates along, or reorganises, the intervening DNA. The distances over which these mechanisms operate vary widely and are constrained both by the mechanism itself and by the mechanical properties of DNA [7–10].

The most abundant of these mechanisms is silencing by nucleoid-associated proteins (NAPs). NAPs bind DNA and mechanically remodel nucleoid architecture through bending, bridging, and lateral filament formation in response to environmental changes, such as pH, temperature, and osmolarity. Histone-like nucleoid structuring protein (H-NS) is the most extensively studied of these, well known as a xenogeneic silencer that suppresses expression of horizontally acquired DNA. H-NS is a homodimeric NAP that, in the presence of AT-rich DNA, forms oligomeric protein-DNA filaments and DNA-H-NS-DNA bridges. These structural changes can ultimately occlude RNAP from promoters or block transcriptional initiation by trapping RNAP within DNA [11,12].

Other repressors instead act through three-dimensional DNA looping, bringing a distal binding site into direct contact with the promoter through space rather than along the DNA. The bacteriophage λ repressor CI is a well-characterised example, looping DNA between operator regions 2.4 kb apart to reinforce lysogenic repression [13]. Repression can also be exerted directly: changes in local DNA supercoiling [8], or the act of antisense transcription itself [9], can alter downstream or convergent promoter activity without direct contact. A further, more targeted route is spreading by sequence-specific DNA-binding proteins that silence neighbouring genes simply by accumulating around their loading site, independent of any dedicated repression function. This has been described for the partitioning proteins of the low copy number plasmids P1 and F, which silence genes flanking their respective centromeric sites as a by-product of their normal partitioning role, albeit by different routes. P1 plasmid ParB spreads outward to form a nucleoprotein filament silencing genes up to 11 kb from *parS* [14,15], whereas F plasmid SopB appears to sequester *sopC*-linked DNA without extending beyond its binding sites [16,17].

KorB, the plasmid-partitioning and repression protein encoded by broad-host-range IncPα plasmids, is itself a member of the ParB protein family described above and might therefore be expected to silence its target promoters simply through passive spreading. However, KorB achieves repression through a distinct route: the action of a DNA-sliding clamp, a mechanism that reconciles decades of competing models for KorB-mediated repression.

The present mini-review traces the history of KorB-mediated long-range gene silencing, from three decades of competing models proposed to explain it to the recent discovery that KorB is a cytidine triphosphate (CTP)-dependent DNA-sliding clamp, a mechanism that, as the present review sets out, is far more than simple passive ParB-family spreading. Particular focus is given to the biochemistry and biophysics driving clamp activation and its subsequent capture by clamp-locking proteins, KorA and TrbA. Finally, the present review closes with the outstanding questions the KorB/KorA/TrbA system raises and their broader relevance to prokaryotic gene regulation, plasmid biology, and the spread of antimicrobial resistance.

## KorB long-range repression: competing models

The *korB* (*kil*-override B) gene was first described in 1982 for its ability to control the lethal *kil* operon of the RK2 plasmid [18]. In the decades since, KorB has proven to play a far broader role in plasmid biology; it is essential both for the stable vertical inheritance of plasmid DNA and for global control of gene expression in plasmids of the broad host-range IncPα family, which includes the aforementioned multidrug resistance RK2 plasmid [19–22].

KorB is a member of the ParB-family of proteins. ParB proteins partition chromosomal DNA together with their partner ParA and the *parS* DNA motif [23–27]. KorB performs the same role for RK2, partnering with the ParA homologue IncC and the *O_B_* DNA motif. This KorB-IncC interaction drives plasmid segregation through a diffusion-ratchet mechanism and ensures both daughter cells inherit at least one copy of the *korB*-encoding plasmid [21,28–32].

However, KorB plays an additional role as a long-range silencer, repressing the expression of key plasmid operons across RK2, including the essential replication operon (*trfA*), the conjugative transfer operon (*kilB*/*trbB*), and the host-lethal *kilA*, *kilC*, and *kilE*-associated operons, several of which are additionally co-regulated by KorC, KorE, and KorF [30,33–38]. Twelve discrete KorB-binding (*O_B_*) sites have been mapped across the ∼60 kb IncPα genome, conserved in both sequence and position in the distantly related IncPβ plasmid R751, underscoring how central this regulatory architecture is to the plasmid's biology [30]; a detailed map of the RK2 operons controlled by KorB, KorA, and TrbA is given in [39].

The distance between these *O_B_* sites and the promoters they regulate has long been difficult to reconcile with a simple steric-occlusion model: *O_B_* sites can lie anywhere from a few base pairs to well over a kilobase upstream of the core promoter elements they control, far beyond the reach of a statically bound repressor (Figure 1B) [40]. This discrepancy motivated three decades of competing models, in which KorB was proposed to either polymerise along DNA from a distal *O_B_* site, promote long-range DNA looping to bring *O_B_* and the promoter into direct contact, or act through some combination of the two (Figure 1C) [41].

However, even this combined model left key experimental discrepancies unresolved. Experimental rotation of an *O_B_* site to the opposite face of the DNA helix had little effect on KorB repression, offering no clear support for looping, which should be disrupted by such alterations. More tellingly, insertion of a LacI DNA roadblock between an *O_B_* site and the target promoter substantially reduced KorB repression [41]. This roadblock sensitivity is difficult to reconcile with a looping mechanism, in which KorB and the promoter would be expected to contact one another directly through three-dimensional space, largely independent of an intervening DNA-bound protein, but is instead consistent with a mechanism reliant on continuous engagement with the DNA track. However, simple KorB polymerisation is similarly inconsistent with the data: estimates of cellular KorB abundance (∼1000 per cell) are insufficient to support a continuous nucleoprotein filament spreading kilobase distances from every *O_B_* site simultaneously, particularly given that KorB must also fulfil its role in plasmid partitioning [41–43].

Resolution came from the archetypal ParAB*S* partitioning system. Chromosome segregation had long relied on a similar, unexplained “spreading” phenomenon, in which ParB accumulates over several kilobases around its centromeric *parS* site. This paradox was resolved by the discovery that ParB binds and hydrolyses CTP. CTP binding drives ParB to close around DNA as a self-loading clamp that escapes the high-affinity *parS* site and diffuses away via 1D diffusion, rather than polymerising outward as a continuous nucleoprotein filament [44–50]. It was this seminal work that led the field to revisit the role of CTP in both KorB plasmid maintenance and, more importantly, its additional role as a transcriptional repressor [40].

## The sliding clamp

The KorB protein comprises an N-terminal intrinsically disordered IncC-interacting motif, an N-terminal CTPase domain (NTD), a central DNA-binding domain (DBD), a long flexible linker, and a C-terminal dimerisation domain (CTD) [30,40]. The KorB-IncC (ParB-ParA) interaction is thought to drive plasmid segregation through a diffusion-ratchet mechanism analogous to that described for other ParAB*S* systems [51,52]. However, this model alone supplies no directional force and has been extended by the DNA-relay model, in which DNA-bound ParA-ATP dimers harness the elastic dynamics of the nucleoid to relay the partition complex across a ParA gradient [53]. The ParB-ParA interaction itself has since been shown to require closure of the ParB clamp [54], the same conformational switch that initiates sliding; whether the IncC-interacting motif is dispensable for gene regulation therefore remains unclear.

KorB engages DNA in two mechanistically distinct steps [45–48]. Initial, sequence-specific loading occurs via the central DBD, which contains a helix-turn-helix motif that recognises the palindromic *O_B_* operator sequence (TTTAGC^G^/_C_GCTAAA) [30,40]; this loading step occurs independently of CTP, and KorB shows no detectable affinity for DNA lacking an *O_B_* site [40]. Concomitant binding of *O_B_* DNA and CTP triggers closure of the clamp: CTP-bound NTDs from opposing KorB protomers dimerise [40,45,46,50], displacing the DBD's DNA-binding surface from the operator and forcing the duplex down into a lumen bounded above by the engaged NTDs, below by the constitutively dimerised CTD, and at the sides by the linkers of the homodimer (Figure 2) [40,48]. This conformational switch forces KorB off its high-affinity *O_B_* site while leaving it topologically entrapped around the DNA [46,47], allowing it to escape and diffuse non-specifically along flanking DNA with a diffusion constant of approximately 1.6 μm^2^/s, around fourfold faster than chromosomal ParB–CTP [40,55]. This diffusion is bidirectional and sequence-independent, with no detectable directional bias, allowing KorB loaded at a single *O_B_* site to populate flanking DNA in both directions, without requiring a directional targeting signal [40].

**Figure 2:**
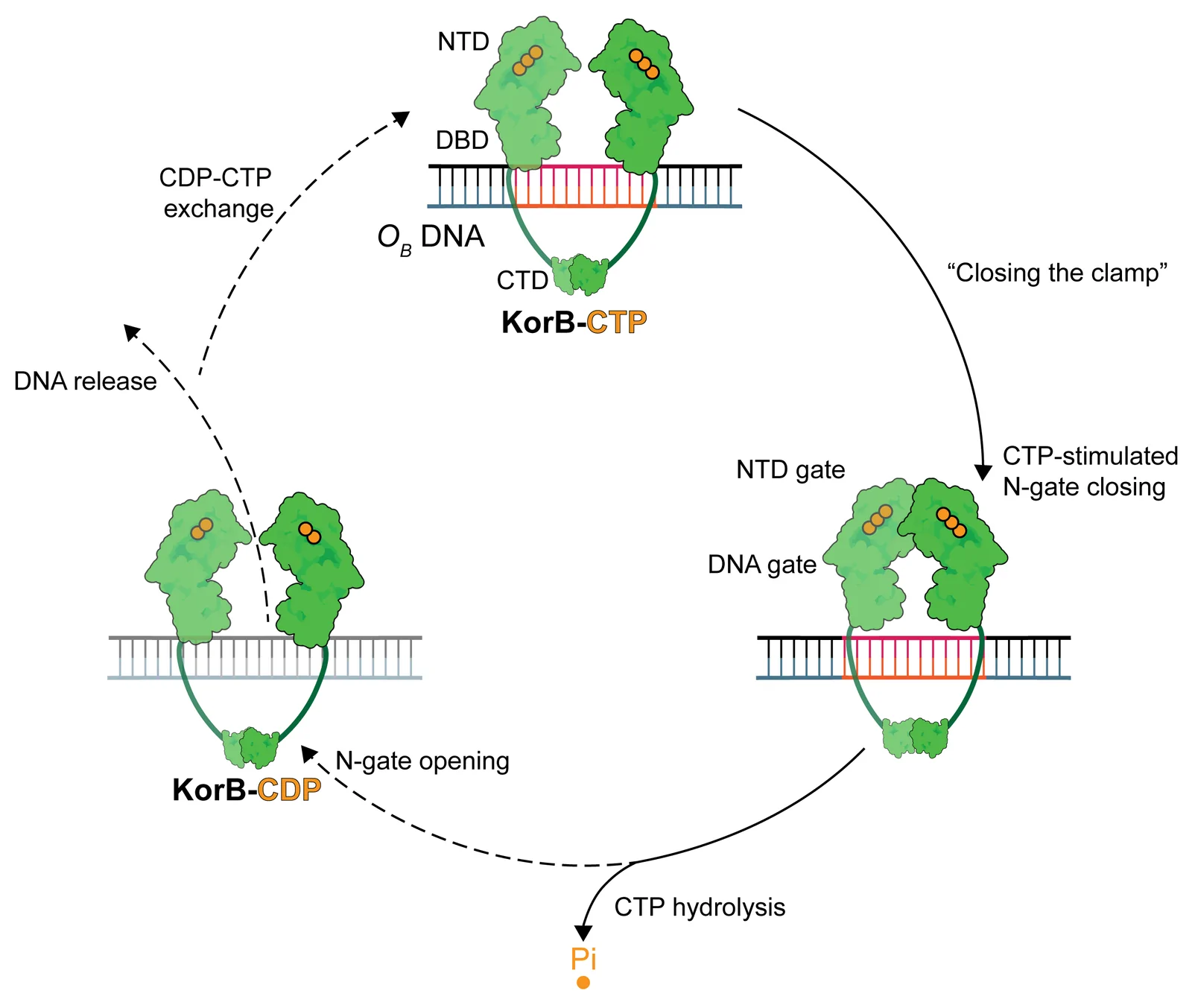
The CTP-driven engagement cycle of the KorB sliding clamp KorB (green) comprises an NTD, a central DBD, and a CTD. Given the low affinity of KorB for CDP relative to CTP and the high intracellular CTP concentration, KorB is likely to be pre-bound to CTP when it encounters *O_B_* DNA, loading sequence-specifically via the DBD in an open state. Engagement of the operator then drives the dimerisation of the two NTDs (“closing the clamp”), closing the NTD gate above the DNA gate and topologically entrapping the DNA duplex. Hydrolysis of CTP releases inorganic phosphate (Pi), leaving KorB-CDP; reopening of the N-gate and release from DNA follow, and exchange of CDP for CTP returns the clamp to a loading-competent state. Solid arrows denote steps demonstrated directly for KorB; dashed arrows denote steps inferred from work on chromosomal ParB proteins.

Biochemically, KorB behaves as a bona fide DNA-stimulated CTPase: it binds CTP with moderate affinity (*K*_d_ ≈ 4.5 μM) and around ten-fold more tightly to the poorly hydrolysable analogue CTPγS (*K*_d_ ≈ 0.4 μM) [40]. In canonical ParB proteins the phosphate-binding motif (GERRxR) is highly conserved, with the glutamate residue essential for CTPase activity but not for CTP binding [48–50]. Despite carrying an alanine at the equivalent position (GARRYR), KorB hydrolyses approximately 60–90 CTP molecules per KorB dimer per hour in the presence of CTP and *O_B_* DNA, a rate comparable to the *Bacillus subtilis* chromosomal ParB-family protein Noc and roughly six-fold faster than *Caulobacter crescentus* ParB [40,56]. Importantly, CTP hydrolysis does not power sliding but does ultimately terminate it. Eventual hydrolysis allows the NTD interface of the KorB homodimer to reopen, releasing the clamp from DNA and imposing an intrinsic lifetime on any individual KorB-DNA engagement [40,49,50].

The traversal of KorB along DNA relies entirely on 1D diffusion, an activity it possesses by virtue of its role in plasmid partitioning rather than one evolved for gene regulation. On its own this is a poor basis for repression: a clamp that samples kilobases of flanking DNA cannot be reliably positioned over any particular promoter when required, and *in vivo* transcriptional reporter assays accordingly indicate only a general two- to four-fold reduction in gene expression, markedly weaker than the ∼200-fold repression achieved by KorA alone (see below) [40]. This is an effect broadly consistent with the passive, non-specific silencing associated with ParB-family protein spreading, rather than evidence of a dedicated repression mechanism [14–17]. Effective site-specific silencing instead requires converting this diffusing, imprecisely positioned clamp into a stationary one. This role is fulfilled by a new class of dedicated clamp-locking proteins, which supply the spatiotemporal control that sliding alone cannot [40].

## Partner proteins

To date two of these clamp-locking proteins have been experimentally validated [39,40]. These are KorA and TrbA, both small homodimeric transcriptional repressor proteins, encoded on the RK2 plasmid [30].

KorA is a site-specific DNA-binding protein that binds as a homodimer to a 12 bp operator sequence, *O_A_*. There are seven *O_A_* sites positioned around the RK2 plasmid, of which five have a corresponding *O_B_* site (*trfA*, *korA*, *kleA*, *klaA*, and *kfrA*) [57]. Importantly, these *O_A_* sites are positioned overlapping the -10 element of the core promoter site. This situates KorA at a location suitable for direct steric hindrance of RNAP binding, the mechanism that it uses itself for medium-level transcriptional repression. However, this also positions it to capture 1D-diffusing KorB, locking the sliding clamp into a stationary position (Figure 3) [40].

**Figure 3:**
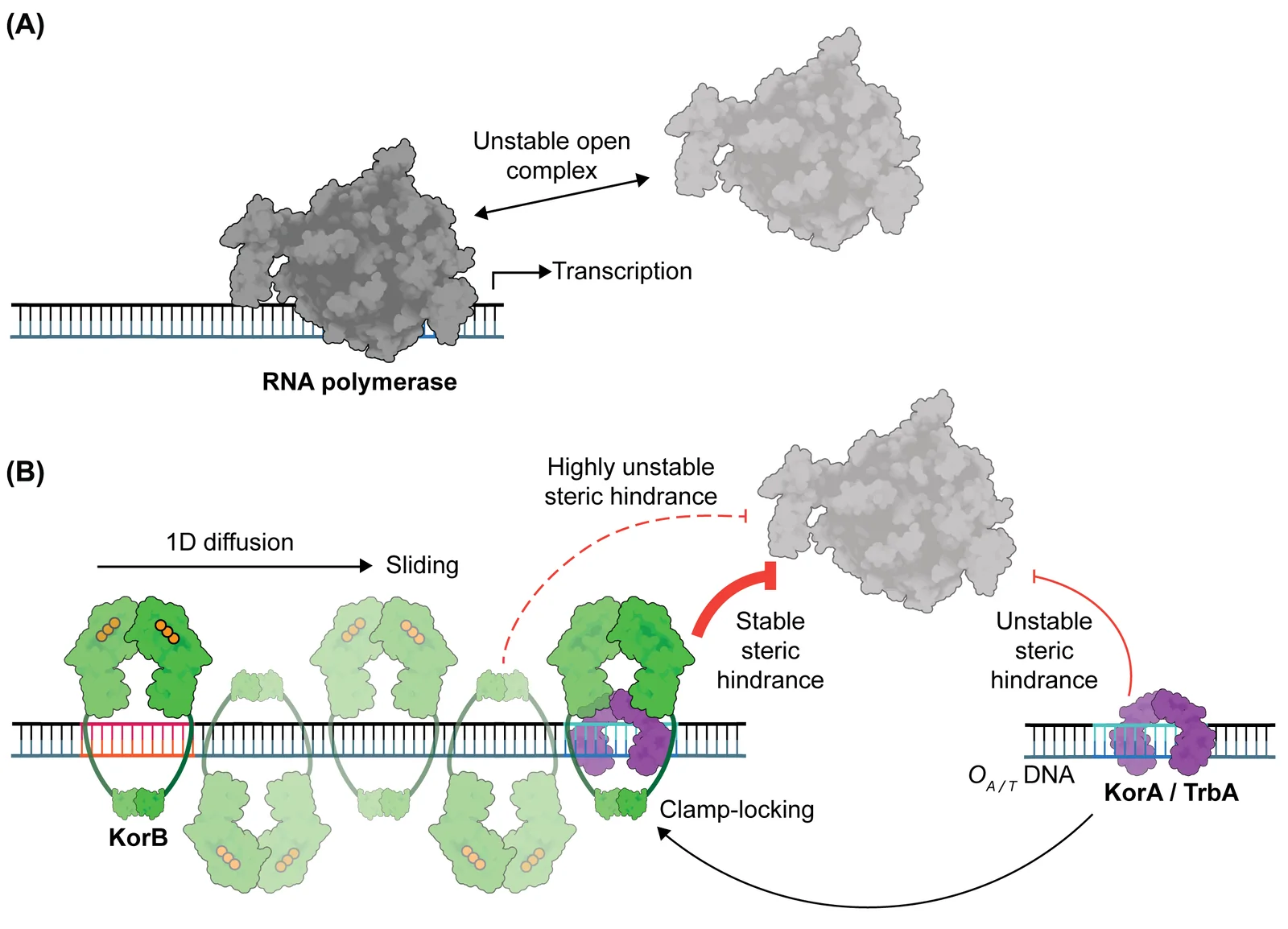
KorA and TrbA convert the sliding clamp KorB into an effective long-range gene silencer (**A**) In the absence of repressors, RNAP (grey) binds the core promoter and initiates transcription. The highly unfavourable discriminator sequences of KorB-regulated promoters give rise to an unstable open complex, so RNAP associates and dissociates frequently, repeatedly leaving the promoter accessible. (**B**) KorB (green), loaded and closed around DNA, undergoes 1D diffusion away from its *O_B_* site, sliding along the DNA duplex. A sliding clamp alone cannot be reliably positioned over the promoter and therefore exerts only highly unstable steric hindrance (dashed red line). KorA or TrbA (purple), bound at its cognate operator (*O_A_*/*O_T_*) overlapping the -10 element, is correctly positioned but only transiently retained, and so produces unstable steric hindrance (thin red line). Upon encountering a bound partner, the sliding KorB clamp is captured through clamp-locking; the resulting stationary co-complex is both larger and longer-lived than either protein alone, producing stable steric hindrance (thick red line) that competitively excludes RNAP from re-forming the open complex.

KorA binds KorB via an interaction interface formed between α10 of KorB, which is located at the C-terminal end of the DBD, just after the helix-turn-helix motif that actually binds *O_B_* DNA, and α5 of KorA, which together with an antiparallel β-sheet forms the dimerisation domain at the C-terminus of KorA. In particular, this interface is reliant on pi-stacking interactions between the aromatic ring of KorA Y84 with the peptide bond of KorB E248-F249 and between the aromatic ring of KorB F249 with the peptide bond of KorA E80-H81. Substitution of either of these aromatic residues (KorB F249 or KorA Y84) with different aromatic residues is tolerated; however, KorB-KorA interaction is abolished if either is substituted for a non-aromatic residue [40].

This clamp-locking interaction has a striking effect on repression efficiency. *In vivo*, KorB alone represses transcription only ∼4-fold and KorA alone ∼200-fold, but when co-expressed at a promoter where *O_B_* lies 1.5 kb upstream and the *O_A_* site overlaps the −10 element, KorA and KorB together achieve up to ∼38,000-fold repression. This is ∼45-fold greater than the product of their individual effects (4 × 200 = 800-fold), indicating true synergy rather than a simple, independent combination of two separate steric hinderers. This cooperativity is entirely lost when the KorA (Y84A) or KorB (F249A) interface mutants are used instead, reinforcing the importance of the aromatic interaction interface [40].

Mechanistically, this cooperation appears to exploit an intrinsic kinetic weakness of the target promoters themselves. KorAB-regulated core promoters on RK2 possess near-consensus -10 and -35 elements but a highly unfavourable discriminator sequence, giving rise to an open RNAP-promoter complex with a half-life of only ∼2 minutes; substituting a favourable discriminator sequence stabilises this complex over 50-fold and reduces the effectiveness of KorA/KorB-mediated repression [40]. These short-lived complexes mean that RNAP vacates the promoter frequently, repeatedly exposing the operator sites to KorA and any KorB clamp sliding past (Figure 3A).

What converts this opportunity into near-absolute repression is that the two proteins stabilise each other once bound. *O_A_*-bound KorA arrests the 1D diffusion of KorB, and KorB-CTP in turn increases the residence time of KorA at *O_A_* approximately 4.5-fold. Both these effects are lost in the KorA (Y84A) and KorB (F249A) interface mutants [40]. Neither protein alone is well suited to the task: KorA binds the right position but is only transiently retained there, while KorB occupies the right region but cannot remain at any one point along it. Captured together they form a stationary co-complex that is both larger and far longer-lived than either repressor alone and which persists across windows in which RNAP would otherwise bind (Figure 3B). Critically, this requires KorB to arrive as a DNA-entrapped clamp rather than simply as a binding partner: although KorA can bind apo-KorB, scrambling the distal *O_B_* site abolishes cooperative repression entirely [40].

This also clarifies what the co-complex does to RNAP. An earlier proposal that KorB might act by contacting the polymerase directly is not supported [58]. No KorB-Eσ^70^ complexes are detectable, and addition of either KorA or KorB to a promoter-bound polymerase instead causes near-complete dissociation of Eσ^70^ from DNA [40]. The stationary KorA-KorB co-complex is therefore best understood as competitively excluding the RNAP holoenzyme from reforming the transient open complex, rather than displacing an already engaged polymerase.

KorA is not the only protein able to interact with KorB and halt sliding. The protein TrbA is paralogous to KorA and has long been proposed to play an analogous role to KorA, forming a tripartite regulatory system within the RK2 plasmid [59,60].

TrbA binds as a homodimer to a distinct operator sequence, *O_T_*, which, like *O_A_*, is typically found overlapping the -10 element of its target promoters. There are five *O_T_* sites on the RK2 plasmid, two of which have corresponding *O_B_* sites (*trbB*, *trfA*) [59]. Recent work has confirmed that TrbA is a true clamp-locking protein for KorB, acting through a mechanism that closely parallels KorA [39,40].

TrbA binds KorB via the same interaction interface used by KorA: helix α7 of TrbA, part of its CTD and equivalent to α5 of KorA, docks onto α10 of KorB. The critical aromatic residue is TrbA F104, equivalent to KorA Y84. Substitution of this residue to alanine abolishes KorB binding, and KorB (F249A) is equally unable to bind TrbA, confirming that KorA and TrbA bind the same interface on KorB in an identical manner [39].

The C-terminal domains of KorA and TrbA are 68% similar (45% identical), while their N-terminal DBDs are more divergent and specify distinct operator sequences, *O_A_* and *O_T_*, respectively, allowing KorB to be recruited to different sets of target promoters depending on which partner is present [39]. However, TrbA binds KorB with lower affinity than KorA (*K*_d_ ≈ 33.5 μM compared with 4.2 μM for KorA) [39,40]. This difference in protein-protein affinity, combined with the range of operator-binding affinities generated by single-nucleotide variation between individual *O_B_*, *O_A_*, and *O_T_* sites, creates an exquisitely tunable hierarchy of regulatory dynamics across the RK2 plasmid. This is important because the KorB/KorA/TrbA system controls operons with very different regulatory requirements. The host-lethal *kil* operons must be held almost completely silent at all times, since even low-level expression is deleterious [18]; replication must be maintained at a level that sustains stable inheritance [29]; and conjugative transfer is required only intermittently. A regulatory system operating at a single strength could not satisfy all three. Graded control instead allows one set of proteins to establish different levels of repression at different promoters, with the strength of repression at each determined by the affinities of its operators and of the partner protein recruited. An operon with a perfect set of operators controlled by KorA and KorB would therefore be predicted to be far more tightly repressed than one with imperfect operators controlled by TrbA and KorB.

## Other ParB-family transcription factors

KorB is not the only ParB-family protein recruited into transcriptional regulation. VirB, of the *Shigella flexneri* virulence plasmid pINV, recognises a *parS*-like operator and binds CTP, yet acts in the opposite manner, relieving H-NS-mediated silencing rather than imposing repression [61–64]. BisD, of the integrative conjugative element ICE*clc*, likewise activates rather than represses, spreading asymmetrically across some 50 kb of ICE DNA in the direction of ICE gene transcription [65].

These proteins differ in how far they have diverged from the ancestral function. VirB has lost partitioning altogether, lacking the N-terminal arginines that mediate ParA contact elsewhere in the family [62], while BisD has gained a domain absent from ParB entirely, through which it engages its partner BisC [65]. KorB is the least diverged, performing both roles with one protein, and the interface KorA and TrbA exploit lies on the α10 of the DBD, a surface the ParB fold already possesses [40].

Most striking, however, is that two of these systems have converged on the same architecture. As with KorA/TrbA and KorB, BisC is bound at its target promoters but cannot act alone. Activation instead requires the arrival of a sliding BisD clamp, which is thought to remodel the BisC-DNA complex and permit RNAP recruitment [65]. The same arrangement has therefore been recruited to opposite regulatory ends. Where the systems differ is in what becomes of the clamp: BisD is proposed to continue sliding onwards towards the next BisC-bound promoter following activation of RNAP, whereas KorB is captured and held, and it is this arrest that allows repression to approach completion [39,40,65]. VirB, which has no comparable partner protein, illustrates the alternative: its regulatory effect diminishes as its binding site is moved further from the promoter it controls [10]. KorB-mediated repression might therefore be better understood not as a repressor hampered by its own sliding, but as the evolutionary co-option of an indispensable partitioning protein into a dynamic regulator of gene expression.

## Concluding remarks and outstanding questions

This clamp sliding-and-locking mechanism reconciles three decades of conflicting models for KorB-mediated silencing. KorB travels kilobase distances along DNA from its *O_B_* loading site; at target promoters, pre-loaded KorA or TrbA arrest the clamp through a conserved aromatic interface, converting a weak and imprecise repressor into a highly effective one. Combined with the kinetic instability of KorB-regulated promoters, this allows repression far beyond what either protein achieves alone.

Several questions remain outstanding. The first is how many clamp-locking proteins exist. KlcB remains a plausible third candidate on RK2, although this is untested, and it is not yet known whether comparable partners are encoded on other IncP plasmids or indeed on unrelated mobile elements. A second question concerns the limits of the mechanism itself. The enlarged lumen of KorB may be what allows a partner protein to be accommodated alongside the DNA duplex, which would suggest that ParB-family proteins with longer linkers are capable of capturing correspondingly larger partners. Finally, it remains unclear whether the roles of KorB are genuinely separable, given that the ParB-ParA interaction requires the same clamp closure that initiates sliding, and this might speak to the evolutionary history of the mechanism in which regulation was likely built atop partitioning.

Repression in this system depends upon a structurally defined protein-protein interface, and interfaces of this kind are, in principle, druggable. Disrupting the KorB-KorA interaction would be expected to derepress the host-lethal *kil* operons that KorB normally silences, thereby destabilising the plasmid from within. Whether this can be achieved in practice remains to be seen, but it is tempting to speculate that such an approach might offer a route to curing resistance plasmids, resensitising their hosts to existing antibiotics. With the antibiotic pipeline unable to keep pace with the spread of resistance, strategies that restore the efficacy of existing drugs, rather than seeking new ones, are likely to become increasingly important.

## Perspectives

- Broad-host-range plasmids such as RK2 are major vehicles for the spread of multidrug resistance, and their propagation depends on coordinated regulation of replication, transfer and partitioning orchestrated largely by KorB.
- KorB is a CTP-dependent DNA-sliding clamp that alone is a poor repressor, relying instead on the clamp-locking proteins KorA and TrbA to capture it at target promoters and convert it into a stationary, highly effective silencer.
- Establishing whether clamp sliding-and-locking operates beyond RK2, and whether the clamp-locking interface can be disrupted pharmacologically, are the principal challenges ahead.

## Open Access

Open access for this article was enabled by the participation of University of Melbourne in an all-inclusive Read & Publish agreement with Portland Press and the Biochemical Society under an agreement with CAUL].

## Abbreviations

CTD: C-terminal dimerisation domain
CTP: cytidine triphosphate
DBD: DNA-binding domain
H-NS: histone-like nucleoid structuring protein
NAPs: nucleoid-associated proteins
NTD: N-terminal CTPase domain
*O_A_*: operator of KorA
*O_B_*: operator of KorB
*O_T_*: operator of TrbA
RNAP: RNA polymerase
